# The nature of π-hole interactions between iodide anions and quinoid rings in the crystalline state

**DOI:** 10.1107/S2052252523000052

**Published:** 2023-01-10

**Authors:** Valentina Milašinović, Vedran Vuković, Anna Krawczuk, Krešimir Molčanov, Christoph Hennig, Michael Bodensteiner

**Affiliations:** a Department of Physical Chemistry, Rudjer Bošković Institute, Bijenička 54, Zagreb 10000, Croatia; b Universität Regensburg, Universitätsstrasse 31, 93053 Regensburg, Germany; c Institut für Anorganische Chemie, Universität Göttingen, Tammanstraβe 4, 37077 Göttingen, Germany; d The Rossendorf Beamline (BM20), European Synchrotron Radiation Facility, 71 Avenue des Martyrs, Grenoble 38043, France; e Institute of Resource Ecology, Helmholz Zentrum Dresden Rosendorf, Bauztner Landstrasse 400, 01328 Dresden, Germany; King Abdullah University, Saudi Arabia

**Keywords:** π-hole interactions, charge transfer, quinone, charge density, Atoms In Molecule analysis

## Abstract

The investigated co-crystal of 3-chloro-*N*-methyl­pyridinium iodide with tetra­bromo­quinone (3-Cl-*N*-MePy·I·Br_4_Q) reveals a π-hole interaction between an iodide anion and a quinoid ring involving an *n* → π* charge transfer. The quinoid ring has a partial negative charge (estimated to be in the range 0.08–0.11*e*) and a partial radical character, which is related to the black colour of the crystals (crystals of neutral tetra­bromo­quinone are yellow). A detailed X-ray charge density study revealed two symmetry-independent bond critical points between the iodide anions and carbon atoms of the ring. Their maximum electron density of 0.065 e Å^−3^ was reproduced by quantum chemical modelling. The energy of the interaction is estimated to be −11.16 kcal mol^−1^, which is comparable to the strength of moderate hydrogen bonding (about −10 kcal mol^−1^); it is dominantly electrostatic in nature, with a considerable dispersion component.

## Abstract 2

Analysis of charge density in a π-hole contact between an iodide anion and tetra­bromo­quinone reveals the nature of the interaction as dominantly electrostatic with a significant dispersion component. The quinoid ring has a partial negative charge (estimated to be in the range 0.08–0.11*e*) and a partial radical character and the energy of the interaction is estimated to be −11.16 kcal mol^−1^.

## 1. Introduction

π-hole interactions, *i.e.* interactions involving areas of electron depletion in π-electron systems, have attracted considerable attention in the fields of supramolecular chemistry and crystal engineering (Frontera *et al.*, 2011 ▸; Wang & Wang, 2013 ▸; Kozuch, 2016 ▸; Angarov & Kozuch, 2018 ▸; Grounds *et al.*, 2018 ▸; Jia *et al.*, 2019 ▸; Kumar Seth *et al.*, 2019 ▸). An electrostatic interaction occurs between the π-hole and an electron-rich group involving a lone electron pair [lone pair–π or lp⋯π interaction (Mooibroek *et al.*, 2008 ▸; Singh & Das, 2015 ▸; Newberry & Raines, 2017 ▸; Kumar Seth *et al.*, 2018 ▸; Angarov & Kozuch, 2018 ▸)] or an anion [anion–π interaction (Gamez *et al.*, 2007 ▸; Schottel *et al.*, 2008 ▸; Frontera *et al.*, 2011 ▸; Wang & Wang, 2013 ▸; Bauzá *et al.*, 2016 ▸; Lucas *et al.*, 2016 ▸; Savastano *et al.*, 2017 ▸)]. The interaction may also involve charge transfer, typically from a lone pair to an antibonding orbital of the π system [*n* → π* interaction (Mooibroek *et al.*, 2008 ▸; Singh & Das, 2015 ▸; Newberry & Raines, 2017 ▸; Angarov & Kozuch, 2018 ▸)]. They are interesting due to their potential application in molecular recognition (Wang & Wang, 2013 ▸; Lucas *et al.*, 2016 ▸; Zeng *et al.*, 2019 ▸) and drug design (Singh & Das, 2015 ▸), and have also been used in crystal engineering (Bauzá *et al.*, 2016 ▸; Kumar Seth *et al.*, 2019 ▸; Bauza *et al.*, 2019 ▸).

**Figure 1:**
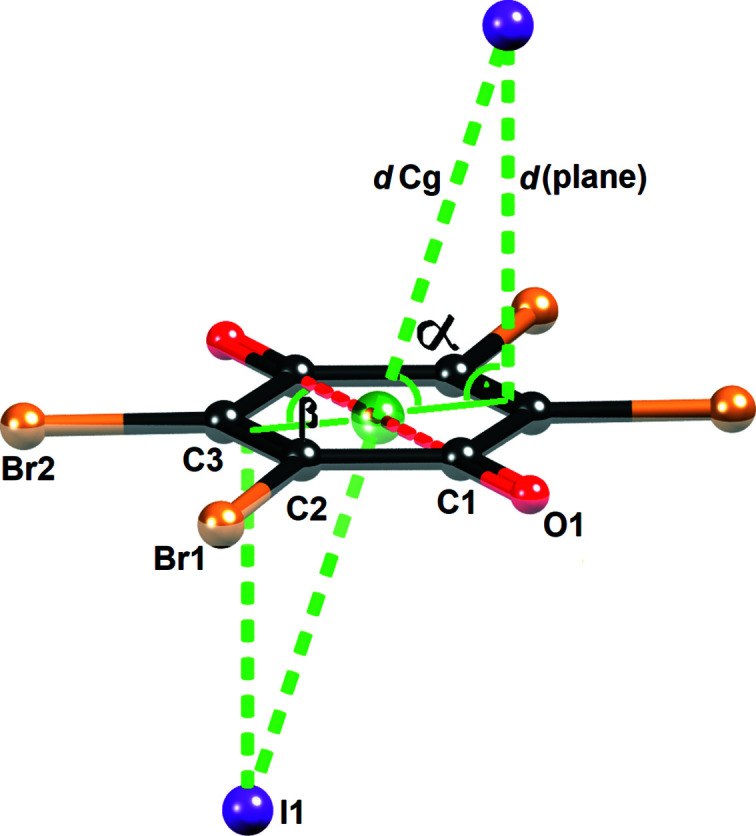
Sandwich-like I^−^⋯Br_4_Q⋯I^−^ unit in **1** with geometric parameters indicated. Symmetry-independent atoms are labelled.

Even though the majority of studies have been carried out on electron-poor aromatic systems, quinoid rings, having electron-depleted carbonyl groups, are more promising acceptors of π-hole interactions (Molčanov *et al.*, 2018 ▸; Kepler *et al.*, 2019 ▸; Wilson *et al.*, 2020 ▸; Milašinović & Molčanov, 2021 ▸). Quinones with four electronegative substituents have especially prominent π-holes (Molčanov *et al.*, 2019 ▸; Vuković *et al.*, 2019 ▸) and their interactions with halide anions often involve a charge transfer, indicated by a colour change of the compound (Molčanov *et al.*, 2018 ▸; Milašinović & Molčanov, 2021 ▸). The common structure motif of the compound under investigation is a sandwich-like group involving two close contacts between a quinone and two halide anions, *X* ^−^⋯*Q*⋯*X* ^−^ (Fig. 1 ▸). The interaction, involving charge transfer is presumably of *n* → π* type, and is often related to the reduction of quinones to semi­quinone radicals. It may be assumed that the ‘sandwich’ is an intermediate in the reduction of a quinone. Owing to its common formation it may be used in crystal engineering.

However, the nature and strength of this quinone–iodide interaction remains elusive, and a detailed study is difficult. The dark colour and opacity of the crystals impede studies that use optical spectroscopy, and the presence of heavy atoms (iodine and bromine) makes quantum chemical study challenging. The strong absorption limits the applicability of X-ray diffraction studies. Our initial work (Molčanov *et al.*, 2018 ▸) on a series of similar compounds used structural characterization that coupled infrared and solid-state NMR spectroscopies and quantum chemical computation [MP2 and periodic density functional theory (DFT)]. The results highlighted the *n* → π* charge transfer and estimated the interaction energy to be 6−10 kcal mol^−1^ (by the MP2 method). However, the degree of charge transfer remained an open issue. Most likely it is quite low (a few percent of an electron), which would be sufficient for a colour change, but not enough to be quantified by spectroscopic methods. A simple analysis of crystal structures using Hirshfeld surfaces (HSs), highest occupied molecular orbitals (HOMOs) and lowest unoccupied molecular orbitals (LUMOs) computed using the *CrystalExplorer* software (Spackman *et al.*, 2021 ▸) and analysis of Voronoi–Dirichlet polyhedra (VDP) (Blatov, 2004 ▸) confirmed this model (Milašinović & Molčanov, 2021 ▸).

**Figure 2:**
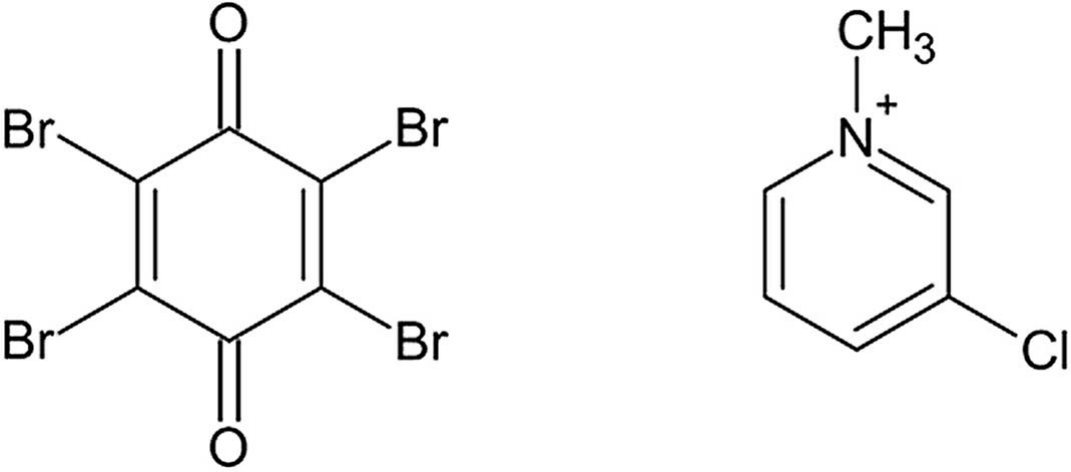
Tetra­bromo­quinone and 3-Cl-*N*-MePy cation.

In this work, we opted for a combined experimental and theoretical charge density study on a model system, a co-crystal of 3-chloro-*N*-methyl­pyridinium iodide and tetra­bromo­quinone [(3-Cl-*N*-MePy)_2_I_2_·Br_4_Q, named **1**, Fig. 2 ▸]. To avoid absorption problems, we used short-wavelength (0.6 Å) high-intensity synchrotron radiation for the X-ray diffraction studies. The analysis of the electron density obtained is further supported by extensive theoretical calculations both in the gas phase and as a crystalline structure. This approach allows us to study in detail the behaviour of molecular orbitals in the areas of crucial interactions, interaction energies as well as provide discussion on the topological analysis of charge density.

## 2. Results and discussion

### 2.1. Crystal packing of **1**

The asymmetric unit of **1** comprises a 3-Cl-*N*-MePy cation, an iodide anion and half of a centrosymmetric Br_4_Q molecule; therefore, the molecular formula is (3-Cl-*N*-MePy)_2_I_2_·Br_4_Q. The easily recognized motif is a sandwich-like I^−^⋯Br_4_Q⋯I^−^ unit (Fig. 1 ▸) which we have identified in similar compounds (Molčanov *et al.*, 2018 ▸; Milašinović & Molčanov, 2021 ▸). The distance from the iodide to the centroid of the quinoid ring is 3.947 Å, the distance to the ring mean plane is 3.727 Å and the angle α between the iodide–centroid axis and the ring plane is 72.0°. The iodide is offset by 1.220 Å approximately towards C2; the angle β defining the direction of the offset relative to the carbonyl–carbonyl axis is 76.8°. The only contact shorter than the sum of van der Waals radii for I and C (3.76 Å) is I1⋯C3 [3.7450 (7) Å].

**Figure 3:**
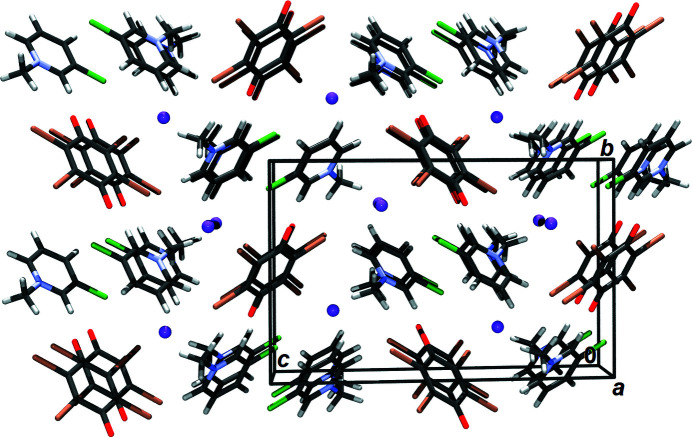
Crystal packing viewed approximately in the 〈100〉 direction. Iodide anions are shown as spheres of arbitrary radii.

Crystal packing can be described as cations inserted between I^−^⋯Br_4_Q⋯I^−^ units (Fig. 3 ▸). Aside from the π-hole contact with Br_4_Q, the iodide forms three halogen bonds, two with bromine from the Br_4_Q molecules and one with a Cl from the cation (Table 1 ▸). It also forms two close contacts (3.70 Å) with N1 and C8 atoms from the cation, which are the result of electrostatic attraction between the cation and anion.

The quinone acts as electron donor of two symmetry-independent halogen bonds (a total of four; Table 1 ▸) and its oxygen atom accepts two weak hydrogen bonds from the cation (Table 2 ▸). A pair of inversion-related cations form a π-stacked pair with antiparallel C–Cl bonds (Fig. S9). The interplanar separation (the ring planes are parallel, so α = 0°) is 3.3879 (3) Å, but the rings are offset by 3.609 Å, so the centroid distance is rather large, 4.9503 (4) Å. This multitude of intermolecular interactions can be ranked according to their strength and importance as (i) cation–anion electrostatic interactions, (ii) iodide–quinone π-hole interactions, (iii) halogen bonding and (iv) stacking interactions between cations.

**Figure 4:**
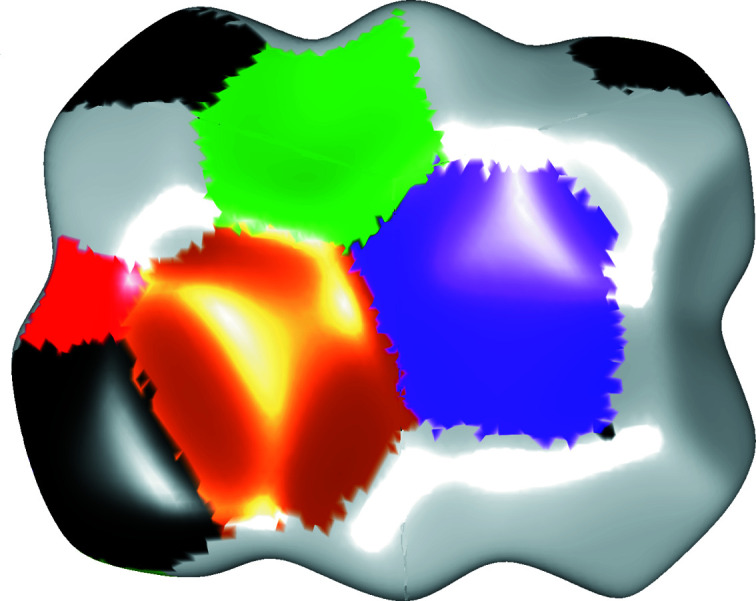
Hirshfeld surface of a Br_4_Q molecule with closest-neighbour atoms colour coded: H – grey, C – black, O – red, Cl – green, Br – brown and I – purple.

The importance of contacts with the iodide anion is illustrated by the HS of the Br_4_Q molecule (Fig. 4 ▸): C⋯I contacts comprise 7.4% of the surface, whereas Br⋯I contacts (representing halogen bonding) comprise a further 7.3%. This is slightly higher than in a previously studied series of co-crystals [where the C⋯I contacts comprised 6–7% of the HS (Milašinović & Molčanov, 2021 ▸)]. The non-localized nature of the iodide–quinone interaction is also noted when the surface of a Br_4_Q molecule is constructed using VDP (Blatov, 2004 ▸): 12 individual faces corresponding to 12 individual C⋯I contacts (six for each symmetry-independent C⋯I interaction, Fig. S10) have a total area of 16.64 Å^2^ or 3.5% of the VDP surface. These contact areas are similar to those found in another series of compounds [typically 6–7% of the HS and 3–3.8% of the VDP surface (Milašinović & Molčanov, 2021 ▸)].

### 2.2. Analysis of intermolecular electron density and iodide–quinone contacts

**Figure 5:**
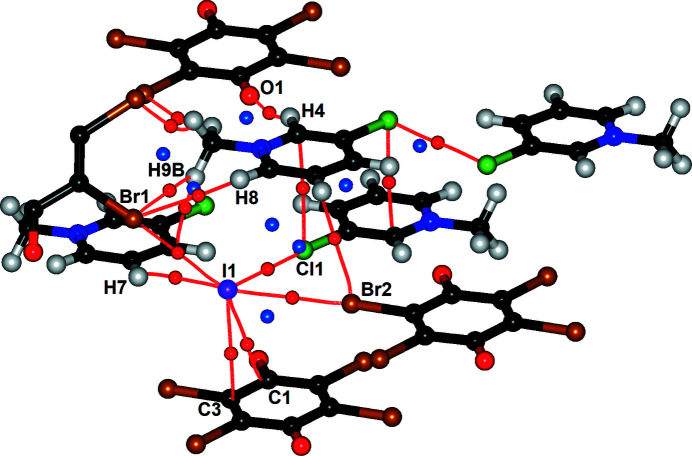
Symmetry-independent intermolecular critical points in **1**. (3, −1) critical points are shown as red spheres and (3, +1) critical points as blue spheres in the interatomic space; bond paths are shown as red lines.

Intermolecular critical points (Fig. 5 ▸, Table 3 ▸) are mostly in agreement with the geometric analysis of crystal packing. There are two (3, −1) critical points between the iodide and the quinoid ring, with respective electron densities of 0.040 and 0.014 e Å^−3^ (Table 3 ▸); the stronger one corresponds to a bond path between I1 and the most electron-depleted atom of the quinone C1 (Fig. 5 ▸). The same critical points have also been reproduced by a periodic DFT model, with somewhat higher electron densities of 0.065 and 0.042 e Å^−3^, respectively (Table 3 ▸). This is consistent with the electrostatic nature of the iodide–quinone interaction, thus the covalent component is likely negligible (see below).

**Figure 6:**
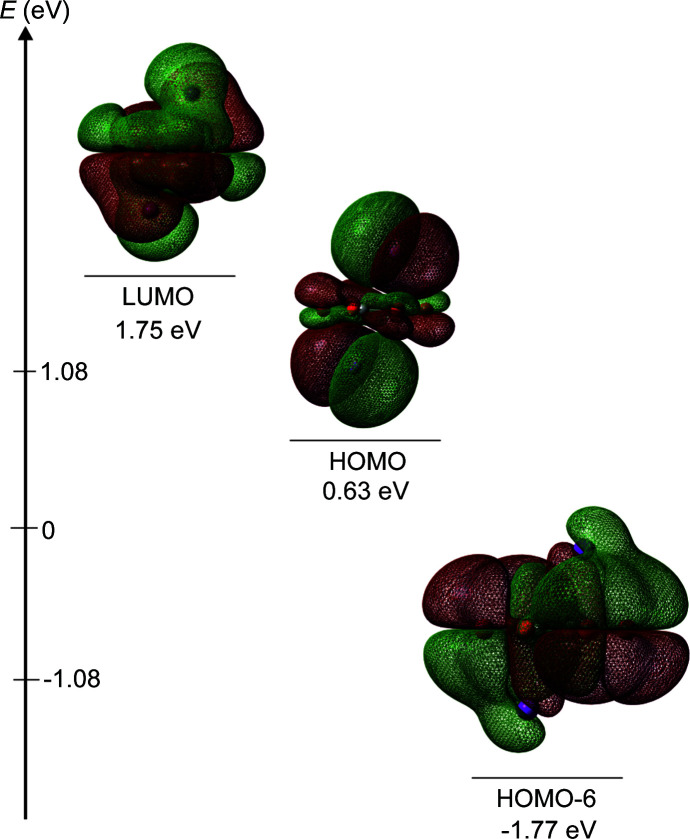
Calculated HOMOs and LUMOs (at an isovalue of 0.001 e au^−3^) for the trimer of a tetra­bromo­quinone molecule and two iodine anions, located at a distance of 3.947 Å from the centroid of the quinoid ring (as observed in the crystal structure).

The analysis of theoretical charges (Table 4 ▸) indicates a partial charge transfer from the iodide to the quinoid ring of −0.077 to −0.109*e*, implying a partial negative charge of the Br_4_Q. This corresponds with the black colour of the crystals and confirms our previous conclusion (Molčanov *et al.*, 2018 ▸). It also provides a more reliable estimate of the degree of charge transfer, as our previous tentative computations were severely overestimated (Molčanov *et al.*, 2018 ▸). Therefore, the interaction is of the *n* → π* type donates electrons into an empty π* (*i.e.* LUMO) orbital of the quinone, which manifests in an overlap of LUMOs in Fig. 6 ▸. Similar behaviour can be observed in the highest binding orbital HOMO-6, where a slight overlap between the iodine orbitals and the quinone ring supports our suggestion that a non-covalent interaction of the π-hole type is present.

**Figure 7:**
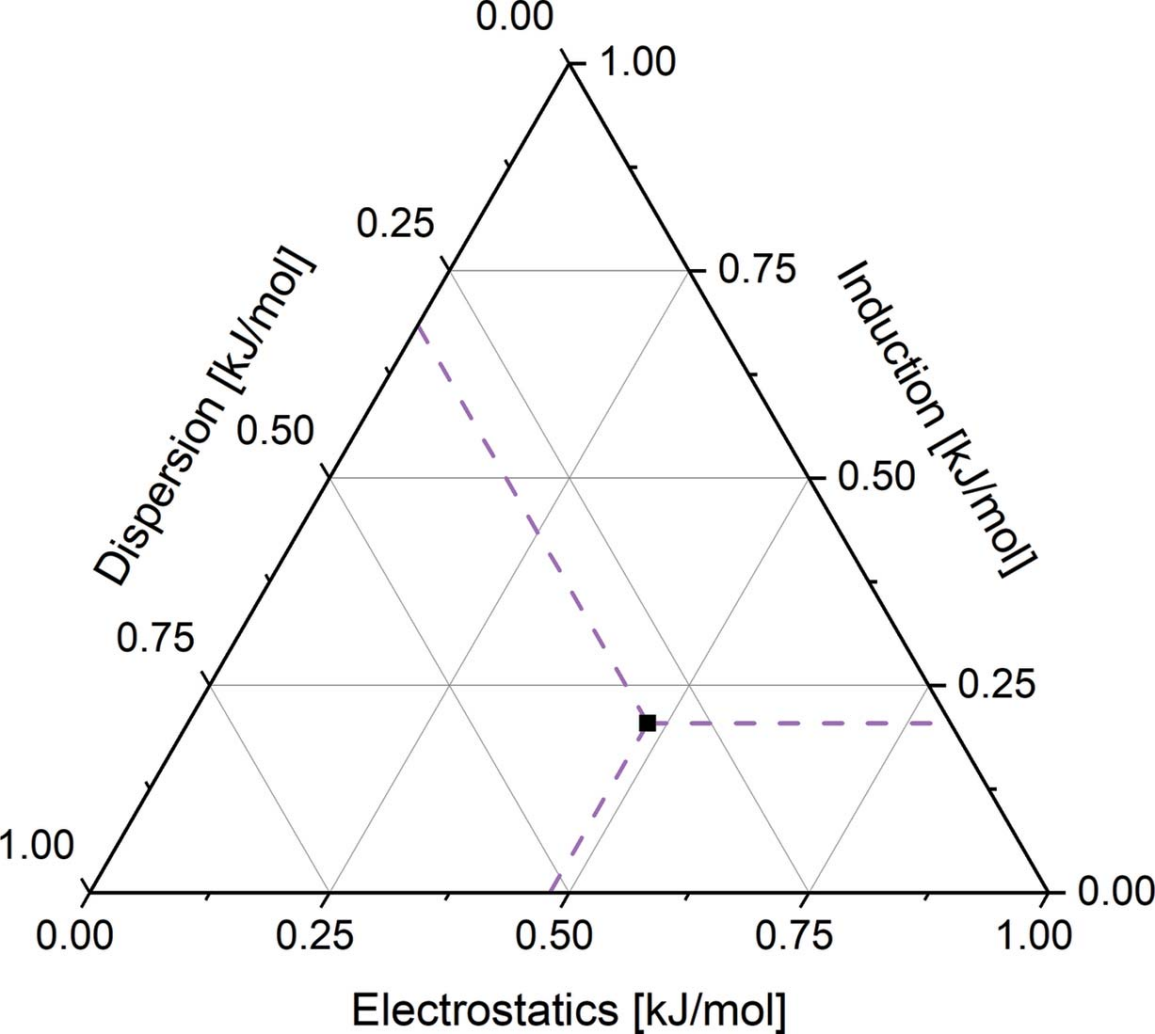
Ternary plot of the SAPT2+3 decomposition of the interaction energy between the Br_4_Q ring and two iodine anions.

The total binding energy calculated for an I^−^⋯Br_4_Q⋯I^−^ unit (Fig. 1 ▸) is −95.36 kJ mol^−1^ and hence for a single I^−^⋯Br_4_Q it is −46.68 kJ mol^−1^ or −11.16 kcal mol^−1^. This value significantly exceeds our previous estimate of 6–10 kcal mol^−1^ (Molčanov *et al.*, 2018 ▸) and is comparable to intermolecular interactions such as hydrogen bonds (Steiner, 2002 ▸) and halogen bonds (Stilinović *et al.*, 2017 ▸; Eraković *et al.*, 2019 ▸). SAPT energy decomposition (Fig. 7 ▸) shows that the dominant component of the total interaction is electrostatic (−86.69 kJ mol^−1^) followed by dispersion (−57.09 kJ mol^−1^).

### 2.3. Other intermolecular contacts

The AIM (Atoms In Molecule) analysis of intermolecular electron density shows that the highest electron density is found in halogen bonds (Table 3 ▸); for C—Br⋯I it exceeds 0.06 e Å^−3^ (0.09 e Å^−3^ in the theoretical model), and it is slightly lower in the C—Cl⋯I bond. This is in agreement with previous studies, which showed that the strength of halogen bonding involving Br as a donor is comparable to hydrogen bonding (Stilinović *et al.*, 2017 ▸) and that it involves a non-negligible covalent component (Eraković *et al.*, 2019 ▸). However, despite higher electron density, the C—Br⋯I halogen bonds are local interactions, whereas the I⋯quinone interaction is non-localized, dispersed between several centres of the Br_4_Q molecule. Therefore, it can be concluded that the halogen bonds are of lesser importance in the crystal packing.

Hydrogen bonding (with the exception of C4—H4⋯O1) is weaker, with a maximum electron density below 0.06 e Å^−3^ (0.11 e Å^−3^ in the theoretical model, Table 3 ▸). Note that two contacts, which satisfy geometric criteria [Table 2 ▸ (see also Steiner, 2002 ▸)] do not have a corresponding (3, −1) critical point, therefore they should not be considered as hydrogen bonds. However, three C—H⋯Br bonds with a *D*⋯*A* distance exceeding 3.9 Å (Tables 2 and 3) have (3, −1) critical points with electron densities of about 0.03 e Å^−3^. This discrepancy between geometric and AIM criteria for weak hydrogen bonding has been noted previously (Milašinović *et al.*, 2020 ▸).

## 3. Conclusions

This work confirmed the nature of the iodide–quinone interaction as a π-hole interaction involving *n* → π* charge transfer. The contact is strongly attractive (its strength and importance in crystal packing are second only to cation–anion electrostatic attraction), with an estimated interaction exceeding −11 kcal mol^−1^, and its dominant component is electrostatic with a significant dispersion contribution. However, a relatively low electron density (not exceeding 0.045 e Å^−3^, Table 3 ▸) found between the iodide and the quinone indicates that the interaction is not localized but dispersed between multiple centres (as shown by the HS and the VDP, Figs. 4 ▸ and S10). The estimated degree of charge transfer between the iodide and the quinone is −0.077 to −0.109 e, consistent with the black colour of the crystals.

Since π-hole interactions between iodide and quinone occur frequently [so far we described more than 20 analogous compounds (Molčanov *et al.*, 2018 ▸; Milašinović & Molčanov, 2021 ▸)], we expect that they can be employed in crystal engineering. However, since the sandwich-like moiety I^−^⋯Br_4_Q⋯I^−^ is probably formed as a stable intermediate in the reduction of the quinone, it can be expected that more electronegative quinones will be reduced to radicals, while the less electronegative ones will not have sufficiently large π-holes. To test the applicability of this interaction in crystal engineering, a larger number of compounds should be tested, including quinones with different substituents (with different electron-withdrawing capabilities) and different nucleophiles (bromine and other halides as well as similar anions such as cyanate, iso­cyanate, thio­cyanate *etc*.).

## 4. Experimental

### 4.1. Preparation and basic characterization

All reagents and solvents were purchased from commercial sources (Merck, Sigma–Aldrich, Kemika), were of p.a. purity and were used without further purification.

Compound **1** (Fig. 2 ▸) was prepared using an analogous procedure similar to previously studied co-crystals (Molčanov *et al.*, 2018 ▸; Milašinović & Molčanov, 2021 ▸): an excess of solid 3-chloro-*N*-methyl­pyridinium iodide was added to a cold (5°C) saturated solution of tetra­bromo­quinone in acetone. Diffraction-quality single crystals were grown overnight.

### 4.2. X-ray diffraction and refinement details

Single-crystal XRD data were collected at the Rossendorf Beamline [ESRF, Grenoble, France (Scheinost *et al.*, 2021 ▸)] equipped with an Si(111) monochromator and two Pd-coated mirrors. The single-crystal data were recorded with a Pilatus3 X 2M detector (Dectris) with an excitation energy of 20000 eV per 0.6200926 Å. The monochromator energy was calibrated against the first inflection of the *K*-absorption edge of an Mo metal foil point, tabulated as 20000 eV. The diffraction measurements were performed in shutterless mode with an angular step size of 0.1° and a counting time of 0.1 s per frame. The detector geometry parameters were calibrated with *PyFAI* (Kieffer & Wright, 2013 ▸) using a powder pattern of the NIST 660b standard LaB_6_. Experimental data were collected using the *Pylatus* software (Dyadkin *et al.*, 2016 ▸) and treated using the *SNBL ToolBox* (Dyadkin *et al.*, 2016 ▸) and *CrysAlis PRO* (Rigaku OD, 2019 ▸).

A total of 131 167 reflections were collected, up to a maximum θ of 40.9° (*d* = 0.475 Å). The multiple integrated reflections were averaged for the space group *P*2_1_/*c* using *SORTAV* (Blessing, 1987 ▸) adapted to the area detector data.

The structure was solved using *SHELXT* (Sheldrick, 2015 ▸) and a spherical-atom model was refined using *SHELXL2017* (Sheldrick, 2015 ▸). Multipolar refinement was carried out versus all reflections *F* ^2^ with the program package *MoPro* (Jelsch *et al.*, 2005 ▸). Halogen atoms were modelled as hexadecapoles, O, N and C as octupoles and hydrogens as dipoles; loose restraints were used for multipoles and exponential κ coefficients of chemically equivalent atoms. Vibrations of halogen atoms were refined as anharmonic using fourth-order Gram–Charlier coefficients. Anisotropic parameters for hydrogen atoms were calculated by the *SHADE3* server (Madsen, 2006 ▸) and kept fixed in the multipolar atom refinement; aromatic C—H bond lengths were restrained to 1.077 (2) Å and methyl C−H bond lengths to 1.083 (2) Å. Geometry and charge-density calculations and analysis of HSs were performed using *MoPro* (Jelsch *et al.*, 2005 ▸); molecular graphics were prepared using *MoProViewer* (Guillot, 2012 ▸) and *CCDC-Mercury* (Macrae *et al.*, 2020 ▸). Crystallographic and refinement data are shown in Table 5 ▸.

Topological bond orders were calculated using the formula (Zarychta *et al.*, 2011 ▸)

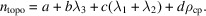

Coefficients *a*, *b*, *c* and *d* were taken from the literature: for C—C bonds, *a* = −0.522, *b* = −1.695, *c* = 0.00, *d* = 8.473 (Howard & Lamarche, 2003 ▸); for C—O bonds, *a* = −0.427, *b* = −0.240, *c* = 0.280, *d* = 6,464 (Tsirelson *et al.*, 2007 ▸); for C—N bonds, *a* = −0.284, *b* = 0.331, *c* = 0.559, *d* = 6.569; (Howard & Lamarche, 2003 ▸); for C—H bonds *a* = −0.153, *b* = 0.481, *c* = 0.983, *d* = 8.087. (Zhurova *et al.*, 2007 ▸).

The analysis of the VDP was achieved using the *Topos PRO* program package (Blatov, 2004 ▸).

### 4.3. Computational details

Gas-phase calculations were carried out in order to obtain more insight into the nature of the quinone–iodide interaction by means of molecular orbitals, atomic charges and interaction energies. Single-point DFT calculations at the B3LYP/def2-SVPD level of theory (Pritchard *et al.*, 2019 ▸) were performed using the *GAUSSIAN 16.C.01* program package (Frisch *et al.*, 2016 ▸). Grimme D3 dispersion correction (Grimme *et al.*, 2010 ▸) was applied in conjunction with the Becke–Johnson damping function and the core electrons for the iodide anions were approximated using pseudopotential functions (Peterson *et al.*, 2003 ▸). Bader charges were obtained with the *AIMAll* software (Keith, 2019 ▸). The SAPT2+3 level was performed with the *Psi4* software (version 1.3.2; Turney *et al.*, 2012 ▸) symmetry-adapted perturbation theory [SAPT (Jeziorski *et al.*, 1994 ▸)] using the same basis set as in single-point DFT calculations.

The choice of a smaller def2-SVPD basis set was found to be a good compromise between the efficiency and accuracy of calculations performed. A benchmark study (Parker *et al.*, 2014 ▸) found the gold standard of SAPT calculations to be the SAPT2+(3)δMP2 using the aug-cc-pVTZ basis set. Unfortunately, this basis set, and the other basis sets included in the benchmark study, did not achieve SCF convergence in the single-point calculations when we tried to predict the molecular orbitals with the experimental structure of the chosen fragment, *i.e.* one Br_4_Q molecule and two iodine anions. Moreover, the triple zeta basis sets would have been rather computationally expensive for SAPT calculations beyond SAPT0. Choosing a basis set that is similar to those in the above-mentioned benchmark study, we found that the def2-SVPD did achieve convergence, was small enough to complete SAPT2+3 calculations in a timely fashion and is accurate enough to describe non-covalent interactions correctly, as already discussed in the literature (Witte *et al.*, 2016 ▸; 2017 ▸). Although we could have extended the single-point calculations that produced the molecular orbital diagrams to a larger def2 basis set, we choose instead to keep the same level of theory for both the molecular orbital calculations and the calculation of the interaction energies. It is also important to point out here that SAPT2+3 calculations are designed to compute energies of dimers. In our studies, we chose a slightly different approach and instead of taking only one iodide anion and quinone ring for consideration, we opted for a trimer, where we treated the Br_4_Q molecule as a single unit, and two iodide anions as a second unit. Such a choice was motivated by the specific crystal packing where indeed the interaction between the quinone molecule and two iodide anions occurs simultaneously. Although such an approach is not commonly used, one may find examples in the literature when more than two units were considered (Yourdkhani *et al.*, 2016 ▸; Steber *et al.*, 2017 ▸).

To further support the discussion on the nature of intra- and intermolecular interactions in the crystalline state, periodic DFT was engaged with the use of the *CRYSTAL17* software (Dovesi *et al.*, 2018 ▸). Calculations were performed on the PBE0/POB-DZVP level of theory (Vilela Oliveira *et al.*, 2019 ▸) applying an additional Grimme’s D3 correction (Grimme *et al.*, 2010 ▸). Atomic coordinates were taken from the X-ray diffraction experiment and were kept frozen during modelling. Periodic wavefunctions obtained in such a way were further used to carry out the topological analysis of periodic electron densities. The QTAIM approach was adopted (Bader, 1990 ▸) using the *TOPOND14* program (Gatti & Casassa, 2017 ▸) integrated with *CRYSTAL17*.

## Supplementary Material

Crystal structure: contains datablock(s) I. DOI: 10.1107/S2052252523000052/ed5028sup1.cif

Structure factors: contains datablock(s) I. DOI: 10.1107/S2052252523000052/ed5028Isup2.hkl

Supporting figures and tables. DOI: 10.1107/S2052252523000052/ed5028sup3.pdf

CCDC reference: 2234264

## Figures and Tables

**Table 1:** Short halogen–halogen distances

	*d* (Å)	C—Br···I (°)	Symmetry operations on Br
C2—Br1⋯I1	3.4960 (1)	173.54 (2)	1 − *x*, −1/2 + *y*, 1/2 − *z*
C3—Br2⋯I2	3.5781 (1)	172.29 (2)	*x*, −1 + *y*, *z*
C5—Cl1⋯I1	3.6225 (2)	168.89 (3)	1 − *x*, 1 − *y*, 1 − *z*
C5—Cl1⋯Cl1†	4.2631 (4)	87.76 (5)	1 − *x*, 1 − *y*, 1 − *z*

† Type II contact.

**Table 2:** Geometric parameters of hydrogen bonds

	*D*—H (Å)	H⋯*A* (Å)	*D*⋯*A* (Å)	*D*—H⋯*A* (°)	Symmetry operations on *A*
C4—H4⋯O1	1.08	2.11	3.1436 (13)	158	1 + *x*, *y*, *z*
C6—H6⋯Cl1†	1.08	2.78	3.8442 (8)	166	−*x*, 1 − *y*, 1 − *z*
C7—H7⋯I1	1.08	3.03	3.8402 (9)	132	−1 + *x*, *y*, *z*
C9A—H9A⋯O1†	1.08 (15)	2.41 (18)	3.4109 (14)	154 (7)	1 + *x*, *y*, *z*
C9—H9C⋯Br2†	1.1 (2)	2.8 (2)	3.8516 (9)	157 (6)	1 − *x*, −1/2 + *y*, 1/2 − *z*
C9—H9A⋯Br1‡	1.08 (15)	3.09 (4)	3.915 (10)	142 (11)	1 + *x*, *y*, *z*
C9—H9B⋯Br1‡	1.08 (11)	3.24 (10)	4.001 (10)	122 (2)	1 − *x*, −1/2 + *y*, 1/2 − *z*
C8—H8⋯Br1‡	1.08	3.21	4.168 (10)	147	1 − *x*, −1/2 + *y*, 1/2 − *z*

† Those found by geometric criteria, but lacking (3, −1) critical points.

‡ Those with (3, −1) critical points but unfavourable geometry.

**Table 3:** Experimental and calculated (italic) intermolecular bonding critical points in the crystal structure of **1**

*A*⋯*B*	*d* (Å)	Electron density (eÅ^−3^) ρ_cp_	Laplacian (eÅ^−5^)	Type	Symmetry operation on *B*
π-hole					
I1⋯C1	4.1060 (7)	0.0141 *0.0424*	0.15 *0.40*	(3, −1)	1 + *x*, −1 + *y*, *z*
I1⋯C3	3.7450 (7)	0.0405 *0.0655*	0.42 *0.67*	(3, −1)	1 + *x*, −1 + *y*, *z*

Halogen bonding					
I1⋯Cl1	3.6225 (2)	0.0600 *0.0594*	0.61 *0.72*	(3, −1)	1 − *x*, 1 − *y*, 1 − *z*
Cl1⋯Cl1	4.2631 (4)	0.0112 *NO BCP FOUND*	0.15	(3, −1)	1 − *x*, 1 − *y*, 1 − *z*
I1⋯Br1	3.4960 (1)	0.0743 *0.0971*	0.73 *0.90*	(3, −1)	1 − *x*, −1/2 + *y*, 1/2 − *z*
I1⋯Br2	3.5781 (1)	0.0637 *0.0813*	0.62 *0.78*	(3, −1)	*x*, −1 + *y*, *z*

Hydrogen bonding					
H4⋯O1	2.11	0.0693 *0.1161*	1.79 *1.69*	(3, −1)	1 + *x*, *y*, *z*
H9A⋯Br1	3.09 (4)	0.0228 *0.0378*	0.35 *0.48*	(3, −1)	1 + *x*, *y*, *z*
H8⋯Br1	3.21	0.0261 0.0290	0.32 *0.24*	(3, −1)	1 − *x*, −1/2 + *y*, 1/2 − *z*
H9B⋯Br1	3.24 (10)	0.0341 *0.0321*	0.39 *0.34*	(3, −1)	1 − *x*, −1/2 + *y*, 1/2 − *z*
H9C⋯Br2	2.8 (2)	0.0290 *0.0607*	0.47 *0.72*	(3, −1)	1 − *x*, −1/2 + *y*, 1/2 − *z*
H7⋯I1	3.8402 (9)	0.0559 *NO BCP FOUND*	0.49	(3, −1)	−1 + *x*, *y*, *z*

C—H⋯π					
H9B⋯C6	2.88 (5)	0.0457 *0.0320*	0.46 *0.36*	(3, −1)	1 + *x*, *y*, *z*

π-stacking					
Cl1⋯C4	3.4361 (7)	0.0452	0.54	(3, −1)	1 − *x*, 1 − *y*, 1 − *z*

**Table 4:** Mulliken and Bader charges calculated in the gas-phase (g) as well as in a periodic system (p)

	Br_4_Q (g)		I^−^Br_4_Q (g)		I^−^Br_4_Q (p)
	Mulliken	Bader	Mulliken	Bader	Bader
C1	0.239	1.055	0.257	1.022	1.061
O1	−0.223	−1.057	−0.281	−1.048	−1.129
C2	−0.054	−0.049	−0.007	−0.054	−0.096
C3	−0.006	−0.047	0.045	−0.054	−0.094
Br1	0.048	0.048	−0.042	0.019	0.073
Br2	0.050	0.050	−0.049	0.028	0.076

I1			−0.923	−0.765	−0.684
Total Br_4_Q	0.000	0.000	−0.077	−0.087	−0.109
Total I^−^B_4_Q			−1.000	−0.852	−0.793

**Table 5:** Crystallographic data collection and charge-density refinement details

Compound	**1**
Empirical formula	C_18_H_14_Br_4_Cl_2_I_2_N_2_O_2_
Formula weight (g mol^−1^)	934.61
Crystal dimensions (mm)	0.12 × 0.09 × 0.09
Space group	*P*2_1_/*c*
*a* (Å)	6.62900 (10)
*b* (Å)	11.07760 (10)
*c* (Å)	17.50330 (10)
α (°)	90
β (°)	97.6870 (10)
γ (°)	90
*Z*	2
*V* (Å^3^)	1273.776 (14)
*D*_calc_ (g cm^−3^)	2.438
μ (mm^−1^)	6.226
θ range (°)	1.59–40.93
*T* (K)	100 (2)
Radiation wavelength	0.62009
Detector type	Dectris Pilatus3 X 2M
Range of *h*, *k*, *l*	–13 < *h* < 13; −23 < *k* < 23; −36 < *l* < 36
Reflections collected	131167
Independent reflections	12766
Reflections with *I* ≥ 2σ	12766
Absorption correction	Analytical
*T*_min_, *T*_max_	0.32961, 1.00000
*R*_int_	0.0289

Spherical refinement	
Weighting scheme	*w* = 1/[σ^2^(*F*_o_2) + (0.0292*P*)^2^ + 0.2885*P*], where *P* = (*F*_o_^2^ + 2*F*_c_^2^)/3
*R* (*F*)	0.0190
*R*_w_ (*F* ^2^)	0.0547
Goodness of fit	1.101
Hydrogen atom treatment	Constrained isotropic
No. of parameters	137
No. of restraints	0
Δρ_max_, Δρ_min_, Δρ_r.m.s._ (eÅ^–3^)	1.745; −1.071; 0.115

Multipolar refinement	
Weighting scheme	*w* = 1/[6σ^2^(*F*_o_^2^)]
*R* (*F*)	0.0164
*R*_w_ (*F* ^2^)	0.0403
Goodness of fit	1.125
H atom treatment	Constrained anisotropic
No. of parameters	709
No. of restraints	477
Δρ_max_, Δρ_min_, Δρ_r.m.s._ (e Å^–3^)	0.488; −0.992; 0.084
