# Supplementary material for: The nature of π-hole interactions between iodide anions and quinoid rings in the crystalline state
Source: IUCrJ. 2023 Jan 10;10(Pt 2):156–63. doi: 10.1107/S2052252523000052 (PMC9980391; doi:10.1107/S2052252523000052)
Supplement: Supplementary file 3 [file m-10-00156-sup3.pdf]

IUCrJ

Volume 10 (2023)

Supporting information for article:

The nature of π-hole interaction between iodide anion and quinoid ring in the crystalline state

Valentina Milašinović, Vedran Vuković, Anna Krawczuk, Krešimir Molčanov, Christoph Hennig and Michael Bodensteiner

S1. Residual densities and other details on refinement

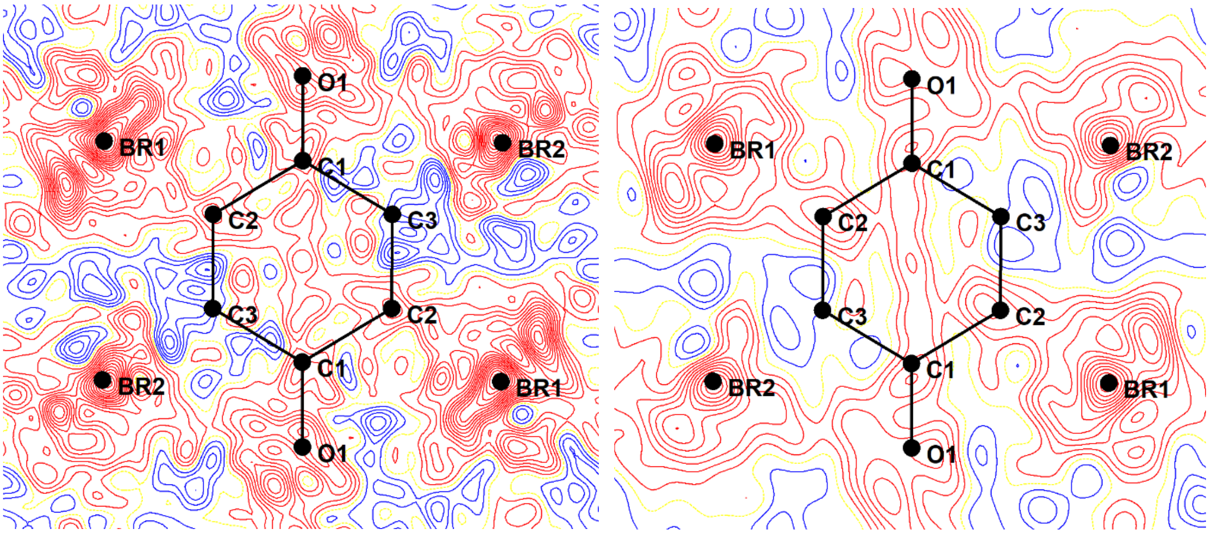

Figure S1 Residual density in the mean plane of Br_4Q with a) all reflections used and b) only low-angle reflections ($\sin\theta / \lambda < 0.7$ Å^{-1}) used. Positive density is shown in blue and negative in red; yellow dotted lines represent zero density. The spacing between contours is of 0.05 eÅ^{-3}.

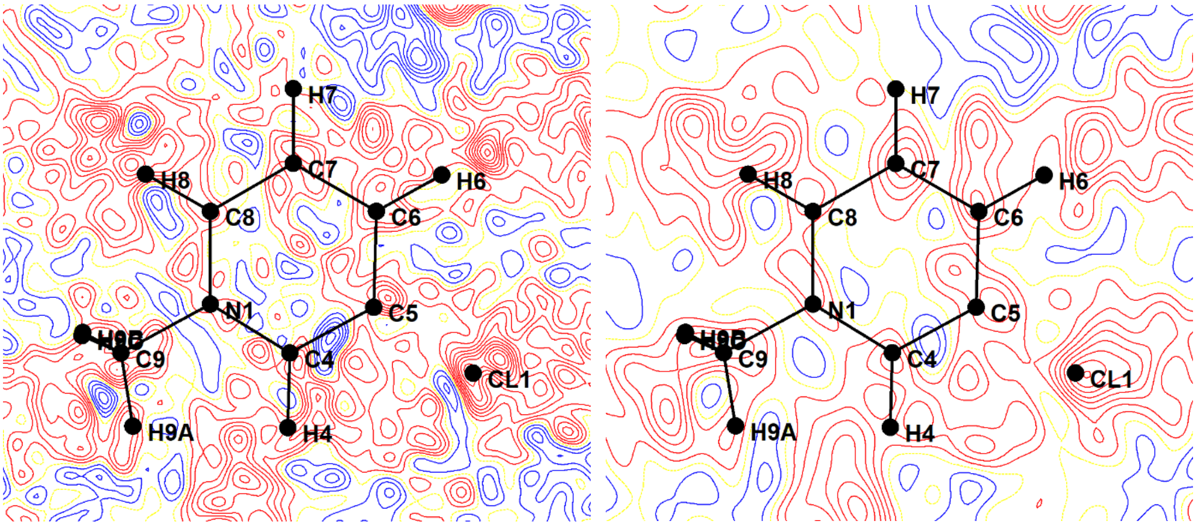

Figure S2 Residual density in the mean plane of 3-Cl-*N*-MePy cation with a) all reflections used and b) only low-angle reflections (s < 0.7 Å^{-1}) used. Positive density is shown in blue and negative in red; yellow dotted lines represent zero density. The spacing between contours is of 0.05 eÅ^{-3}.

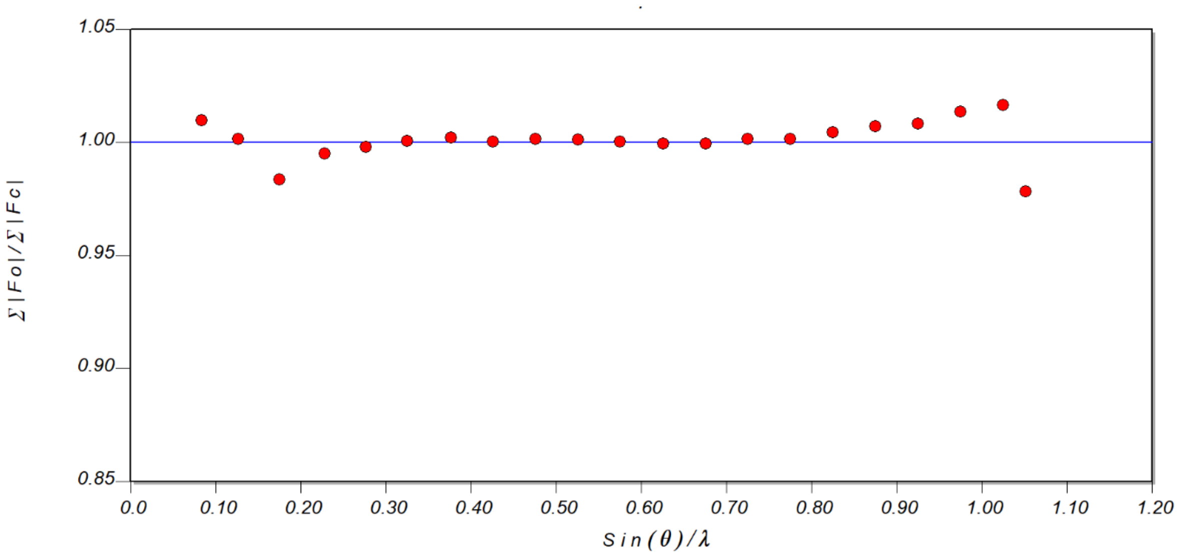

Figure S3 XDRK plot showing the fit of $<Y_{obs}>$ *vs.* $<Y_{calc}>$ as a function of resolution.

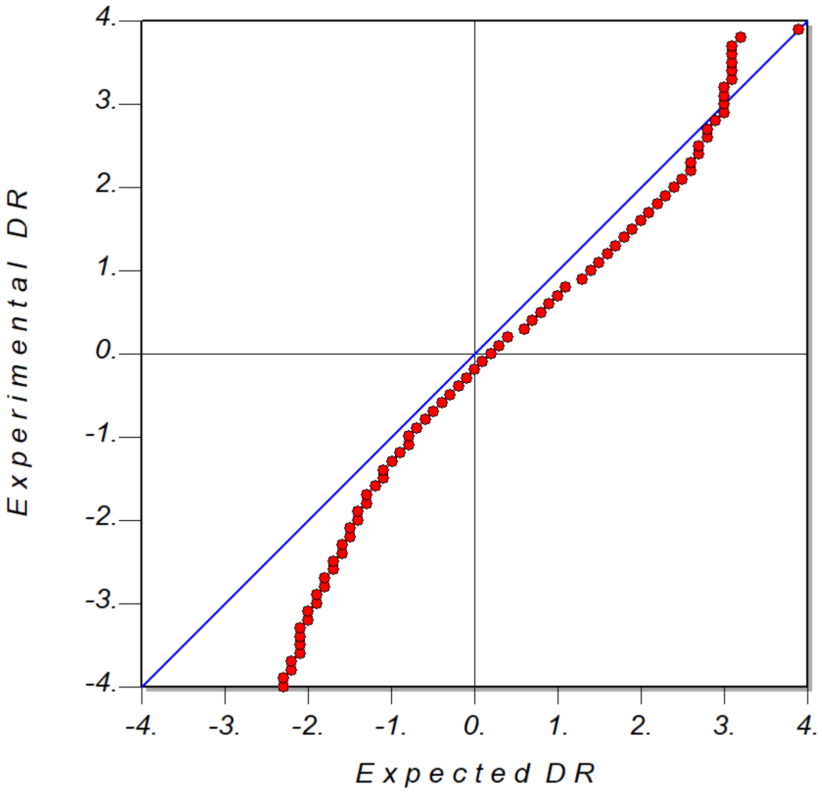

Figure S4 XDRK plot showing the expected and experimental $Y_{obs} - Y_{calc}$ data profile.

S2. ORTEPs, deformation densities, Laplacians and electrostatic potential

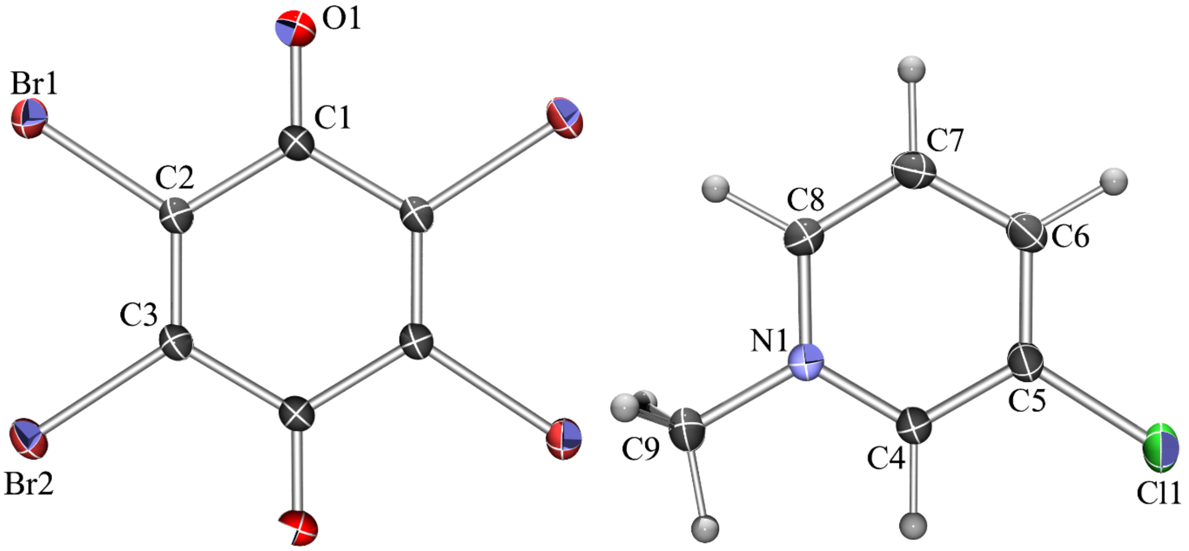

Figure S5 ORTEP-3 drawings of of a) Br_4Q and b) 3-Cl-*N*-MePy cation. Displacement ellipsoids are drawn for the probability of 50% and hydrogen atoms are shown as spheres of arbitrary radii.

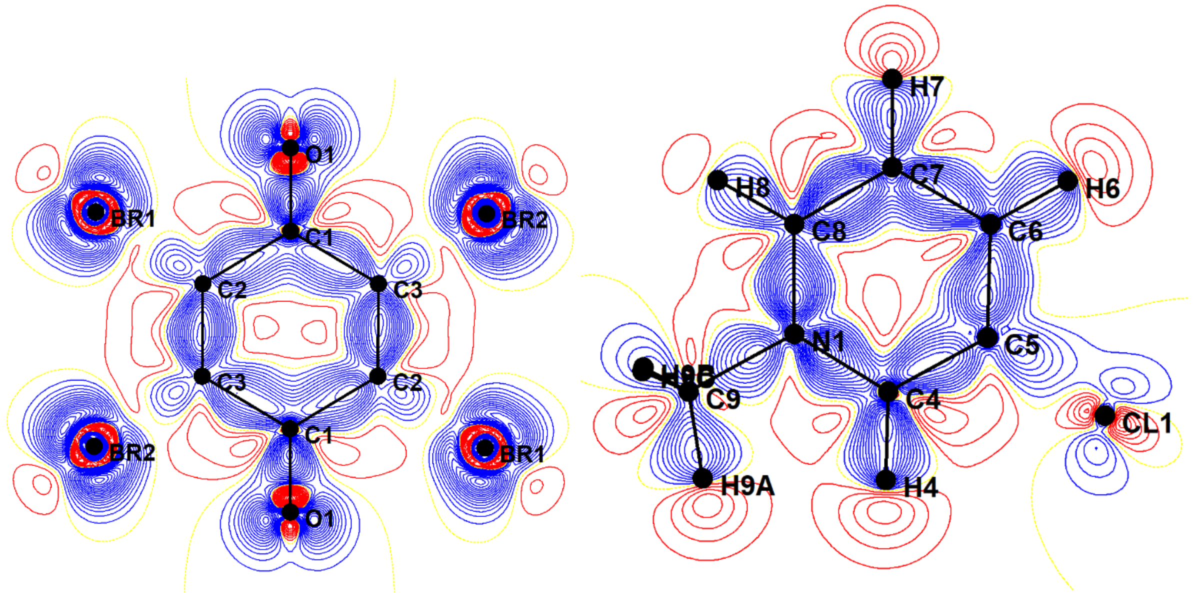

Figure S6 Deformation density maps in the mean planes of a) Br_4Q and b) 3-Cl-*N*-MePy cation. The spacing between contours is of 0.05 e Å^{-3}; the positive density is blue, the negative is red and the zero contour is drawn as a yellow dotted line.

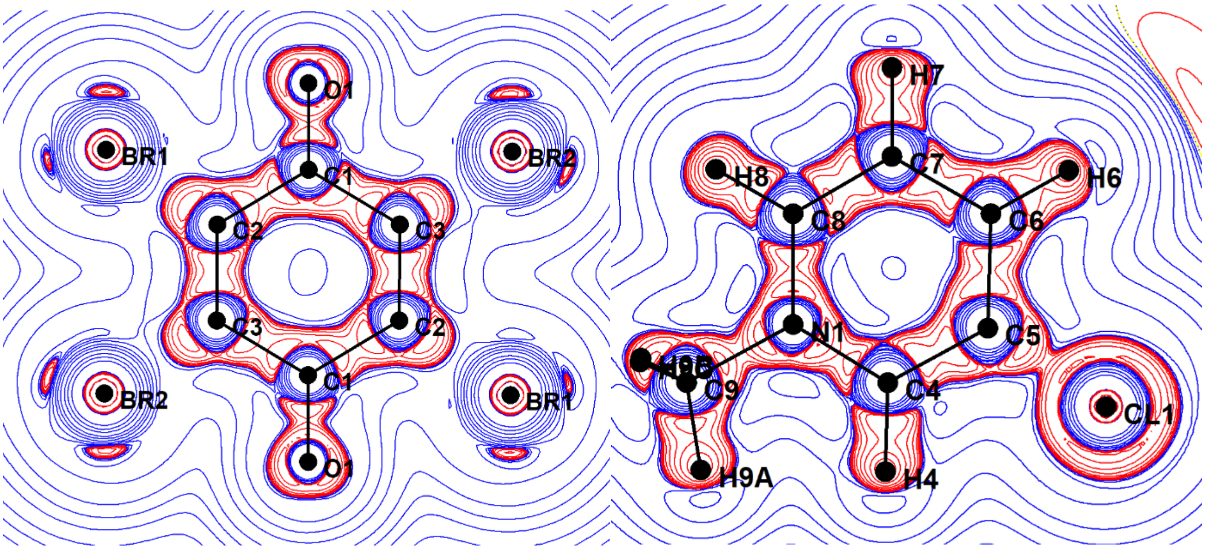

Figure S7 Laplacian of electron density plotted in the mean planes of a) Br_4Q and b) 3-Cl-*N*-MePy cation. The contours are drawn for 2, 4, $8 \cdot 10^n$ e Å^{-5}, $n = -3 \ldots 2$; positive Laplacian is blue and negative is red.

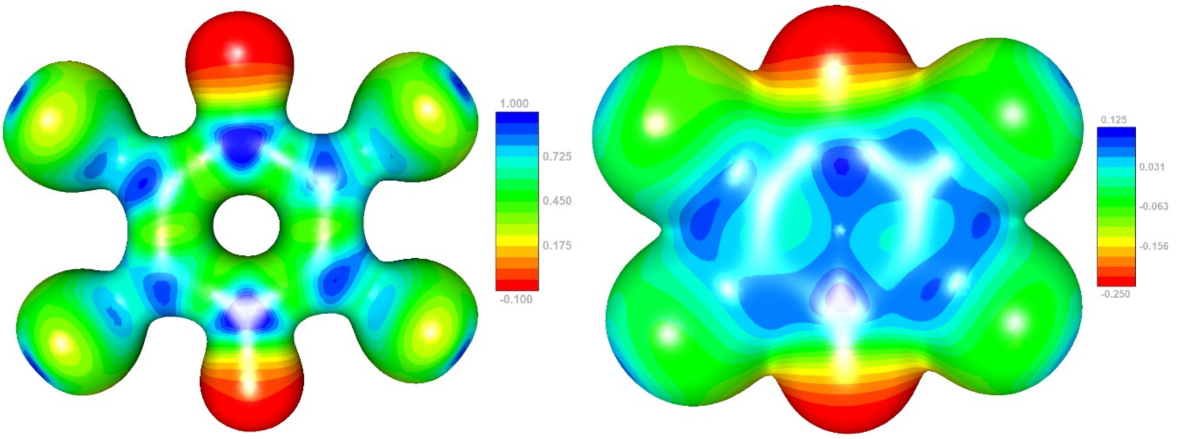

Figure S8 Electrostatic potential of Br4Q molecule plotted onto an electron density isosurface of a) 0.5 e Å^{-3} and b) 0.05 e Å^{-3}.

S3. Other geometric data

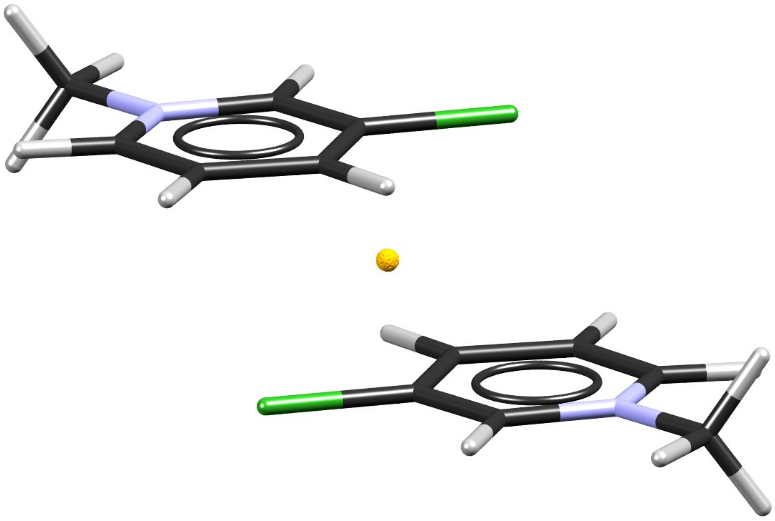

Figure S9 A pair of antiparallel stacked 3-chloro-*N*-methylpyridinium cations related by an inversion centre (shown as a yellow sphere).

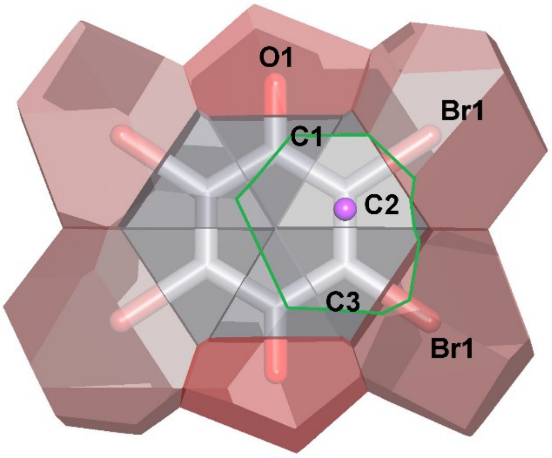

Figure S10 Surface of a Br_4Q molecule comprised of VDPs. Area corresponding to the contact with the iodide (shown as a purple sphere near C2) is highlighted in green.

S4. Topology of electron density

Table S1 Topology of electron density in Br_4Q, derived from electron-density after multipole refinement and periodic calculations (in italic).

Bond	Length (Å)	Electron Density ($eÅ^{-3}$) ρ_{cp}	Laplacian ($eÅ^{-3}$)	Ellipticity	Bond order n_{topo}
C1−O1	1.2130(13)	2.874<br>*2.675*	−29.4<br>*−2.410*	0.17	1.50
C1−C2	1.4909(10)	1.790<br>*1.769*	−12.3<br>*−14.459*	0.10	0.86
C2−C3	1.3462(9)	2.307<br>*2.201*	−22.5<br>*−19.038*	0.27	1.65
C3−C1	1.4898(10)	1.840<br>*1.773*	−13.1<br>*−14.459*	0.09	0.90
C2−Br1	1.8778(7)	1.001<br>*1.131*	1.7<br>*−4.097*	0.26	
C3−Br2	1.8767(6)	0.990<br>*1.134*	1.7<br>*−4.097*	0.13	

Table S2 Topology of electron density in 3-Cl-*N*-MePy cation, derived from electron-density after multipole refinement and periodic calculations (in italic).

Bond	Length (Å)	Electron Density ($eÅ^{-3}$) ρ_{cp}	Laplacian ($eÅ^{-3}$)	Ellipticity	Bond order n_{topo}
N1−C4	1.3497(9)	2.256<br>*2.124*	−21.7<br>*−15.905*	0.16	1.30
N1−C8	1.3469(10)	2.356<br>*2.132*	−26.0<br>*−15.423*	0.21	1.29
N1−C9	1.4745(11)	1.749	−10.6	0.04	1.04

		1.603	*−10.603*		
C4−C5	1.3833(10)	2.049	−18.5	0.15	1.29
		2.103	*−18.315*		
C5−C6	1.3924(11)	2.121	−17.0	0.18	1.47
		2.054	*−17.592*		
C6−C7	1.3889(12)	2.119	−19.9	0.19	1.29
		2.041	*−17.110*		
C7−C8	1.3865(12)	2.272	−21.1	0.17	1.50
		2.083	*−18.074*		
C5−Cl1	1.7262(7)	1.401	−1.3	0.08	
		1.346	*−6.266*		
C4−H4	1.08	1.820	−19.2	0.09	0.90
		1.892	*−22.412*		
C6−H6	1.08	1.701	−16.9	0.03	0.99
		1.869	*−21.201*		
C7−H7	1.08	1.681	−19.8	0.09	0.80
		1.864	*−21.063*		
C8−H8	1.08	1.550	−13.6	0.12	0.82
		1.890	*−21.914*		
C9−H9A	1.08(15)	1.725	−16.8	0.14	0.96
		1.892	*−20.966*		
C9−H9B	1.08(11)	1.443	−7.8	0.16	0.92
		1.879	*−20.484*		
C9−H9C	1.08(19)	1.692	−16.8	0.15	0.93
		1.885	*−20.868*		
